# In Pursuit of Recovery: A Comparative Study of Stakeholder Perspectives on Outcomes of People with Psychosis

**DOI:** 10.1007/s10597-024-01399-9

**Published:** 2024-12-10

**Authors:** Violet van Dee, Wilma Swildens, Hugo G. Schnack, Wiepke Cahn

**Affiliations:** 1 Brain Center, Department of Psychiatry, University Medical Center Utrecht, Utrecht University, 3508 Utrecht, GA The Netherlands; 2 Altrecht Institution for Mental Health Care, Utrecht, The Netherlands; 3 Inholland University of Applied Sciences, Amsterdam, The Netherlands; 4 Institute for Language Sciences, Utrecht University, Utrecht, The Netherlands

**Keywords:** Schizophrenia, Recovery-oriented care, Service users, Informal caregivers, Healthcare professionals

## Abstract

There is no consensus on the definition of recovery of people with psychosis. This may be attributed to the broad concept including clinical, functional and personal recovery domains and the diverse interests of stakeholders involved. We hypothesized that service users, informal caregivers and healthcare professionals would exhibit differences in prioritization, reflecting varying roles and viewpoints. Through an open online anonymous questionnaire, stakeholders shared their perspectives on most important aspects of clinical, functional and personal recovery and on facilitators and barriers of recovery. Stakeholder collaboration in study design and interpretation enhanced the study’s robustness. The answers on open questions of respondents were categorized for statistical analyses to compare the answers between respondent groups on the different aspects of recovery. 226 stakeholders participated. While commonalities prevailed, distinctions emerged in recovery domain priorities. Service users and informal caregivers accorded equal importance to clinical, functional and personal recovery, whereas healthcare professionals emphasized clinical recovery. Regarding functional recovery, service users seemed to focus on practical issues while informal caregivers addressed the underlying causes. Disparities were notable in the role of healthcare that was considered as both a facilitator or a barrier to recovery, with informal caregivers prioritizing aspects of healthcare more frequently as most important than healthcare professionals. While commonalities prevailed, we identified several differences in prioritizing recovery domains among stakeholder groups. Awareness and understanding of these differences is crucial for effective communication and collaboration between stakeholders in recovery-oriented healthcare. The study underscores the need for ongoing dialogue between stakeholders.

**Supplementary Information:**

The online version contains supplementary material available at 10.1007/s10597-024-01399-9.

## Introduction

Psychotic disorders, with a lifetime prevalence of 1% (Moreno-Küstner et al., 2018), significantly impact various aspects of life. Beyond challenging symptoms, people with (a history of) psychosis experience challenges in social, societal and personal functioning. Understanding the outcomes of people with psychosis requires a comprehensive approach with attention for different domains of recovery such as clinical, functional and personal recovery. Clinical recovery is defined as the reduction or remission of symptoms, functional recovery as improvement in occupational, social and adaptive functioning, and personal recovery as the development of personally valued goals, meaning and identity (Chan et al., 2018). However, the lack of consensus on outcomes definitions persists, as exemplified by a survey of 10,000 trials in schizophrenia, which employed 2194 different outcome scales (Miyar & Adams, 2013). The wide variety of outcome measures hinders the comparability of study results and the translation of research findings into individual patient care (van Dee et al., 2023; Huxley et al., 2021; Keeley et al., 2015).

This lack of consensus may be attributed to the diverse interests of stakeholders involved. Historically, healthcare professionals primarily focused on outcomes determined by symptomatic relief, equating recovery with a return to the status before the onset. Over time, the deinstitutionalization of mental healthcare and the emergence of the recovery movement shifted the focus to community functioning and self-determination (Maybery et al., 2021; Turner, 2023). Today, recovery-oriented healthcare places the diverse needs of people with psychosis at the center of treatment, recognizing their unique experiences, initiatives, desires, and goals (Turner, 2023). From this perspective, recovery is an ongoing, highly personalized, non-linear trajectory (Roe et al., 2007). While the philosophy of recovery-oriented care enjoys widespread support, variations persist in the prioritized domains of outcomes. A study investigating remission as perceived by triads of service users, family members and health care professionals showed that in only 18% all three agreed in their assessments of remission (Karow et al., 2012). Healthcare professionals most often emphasize clinical recovery, prioritizing symptom reduction, fewer hospitalizations, and treatment adherence (Karow et al., 2012; Savill et al., 2024; Sood et al., 2022). Informal caregivers more often emphasize the importance of clinical and functional recovery, prioritizing symptom reduction as well as psychosocial outcomes like having relationships, reduced stress, daytime activities and becoming more independent (Gopal et al., 2020; Sood et al., 2022; Vera San Juan et al., 2021). People with psychosis more often emphasize both functional and personal recovery, prioritizing recovered capacity for work, subjective well-being, being accepted and self-respect (Bridges et al., 2013; Sood et al., 2022; Vera San Juan et al., 2021). To collaborate effectively, stakeholders must be aware of the differences in perspectives and the potential underlying causes. The differences in perspectives may be linked to the culture and paradigm of mental healthcare in which, according to all three stakeholder groups, both ‘stigma’ and ‘not speaking the same language’ may act as barriers to equal participation (Landeweer et al., 2017).

Several gaps were identified in existing studies focusing on stakeholder perspectives on recovery. First, many studies focus on a selection of stakeholder groups and/or domains of outcome. Second, many studies use focus groups with small selections of participants. Third, the wide variety of research designs used makes data pooling and comprehensive conclusions challenging. We found only three studies that compared the perspectives of service users, informal caregivers and healthcare professionals; the first investigated the desired outcome of a treatment program for ‘difficult to treat’ schizophrenia with focus groups (Sood et al., 2022), the second investigated whether triades of stakeholders considered the individual service users in remission (Karow et al., 2012), and the third study aimed to propose a theoretical explanation of the recovery process using the Grounded Theory approach (Noiseux et al., 2010). Fourth, there is a need for the involvement of service users and caregivers in the research design in this field, to make sure the right questions are asked and the results are interpretated correctly (Trujols et al., 2013).

To add knowledge to these gaps and gain contemporary insights on stakeholder perspectives on recovery of people with psychosis, we conducted a questionnaire-based research project. Service users, informal caregivers and healthcare professionals were involved in the development of the questionnaire and interpretation of results. Our primary research goal was to gain insight in the aspects that are considered most important by service users, informal caregivers and healthcare professionals to achieve clinical, functional and personal recovery and to identify similarities and differences in perspective between these groups. Our secondary research goal was to identify the priorities, similarities and differences in perspective on facilitators and barriers of recovery between stakeholder groups.

## Methods

### Study Design

Three groups—service users with (a history of) psychosis (SUs), informal caregivers (ICs) and healthcare professionals (HCPs) of service users with (a history of) psychosis—reported their views on different aspects of recovery in an anonymous online survey. The study had a mixed-method design, combining elements of quantitative and qualitative research methods to answer the research question. The answers to open questions of respondents were categorized for statistical analyses to compare the answers between respondent groups on the different aspects of recovery. The Ethics Committee of the University Medical Centre Utrecht gave permission for this study (protocol number 21-068-C).

### Respondents and Settings

The invitation to participate was spread via LinkedIn, online forums for service users, service user associations, associations for informal caregivers of people with psychosis, recovery colleges (Thériault et al., 2020) and organizations for mental healthcare. The invite comprised a link to the online secure survey software program Castor EDC (Electronic Data Capture) (Castor, 2019). Informed consent was required before respondents could start filling in the survey. The survey was conducted from February 2021 to January 2022. Eligibility criteria were having experience with psychosis (regardless of diagnostic classification) as a service user, IC or HCP and being able to read and write Dutch. Service users, ICs and HCPs could participate independently, they did not have to be related to each other.

### Survey

The survey was developed with, and pretested by service users, an IC and HCPs to ensure that the right questions were asked and that questions were clear.

The first part of the survey consisted of closed-ended questions. First, respondents’ demographic characteristics were collected, including age, sex, level of education and being spiritual. In addition ICs were asked about their relationship with the service user and the frequency of contact. HCPs were asked about their profession, the treatment setting they were working in and years of work experience in mental healthcare. Second, for service users and ICs, service user characteristics (for service users about themselves, for ICs about their loved one) were collected, including demographic information, DSM-classification, treatment setting and recovery phase. Recovery phase was divided into four categories; being overwhelmed by, struggling with, living with and living beyond the disease (Gestel-Timmermans et al., 2012; Spaniol et al., 2002).

In the second part of the survey, that consisted of open-ended questions, respondents were asked which aspects of outcome were deemed most important to them. To guide the thinking process, respondents were asked to answer this question regarding the following domains of outcome; clinical recovery, functional recovery (split up into independent living, social participation, daytime activities and other aspects) and personal recovery. For each domain of outcome respondents were asked; *‘In your opinion, which aspects of [domain] are most hindering to you/your loved one/your service users? On which aspect of [domain] should the treatment be focused primarily?*’ For each domain of outcome respondents could provide and prioritize a maximum of five answers. Respondents had the option to skip specific questions. Unanswered questions were categorized under a ‘no answer’ response category, rather than being treated as missing data. At the end of this part of the questionnaire, respondents were asked to sort their own answers to all questions from most important to least important.

In a third part of the survey, about factors that influence recovery, respondents were asked to name and prioritize a maximum of five facilitators of recovery, barriers of recovery, and personal strengths of service users that contribute to recovery.

### Data Analysis

The answers of respondents were exported as data strings from Castor EDC, resulting in an excel based data file with answers on both the closed-ended and open-ended questions. All respondents that provided at least one answer in the second (domains of recovery) or third (facilitators and barriers) part of the questionnaire were included for analysis.

For the content analysis of the answers provided for each open-ended question, coding categories were established by two researchers (VvD and WS). The researchers primarily used conventional content analysis, allowing categories to be derived directly from the data for each open question (Hsieh & Shannon, 2005). When this resulted in an excessive number of small, narrowly defined categories, a directed content analysis strategy was applied. In directed content analysis, existing theory and prior research is used to establish coding categories (Hsieh & Shannon, 2005). All answers were coded independently by two researchers (VvD and WS), according to the established categories. Differences in judgement were settled in consensus meetings.

The statistical analysis was done using R version 4.3.1 (2023-06-16). The characteristics of respondent groups were compared using Fisher’s exact tests for the comparison of proportions and ANOVA for continuous data.

To answer our primary and secondary research questions, for each open question (all domains of outcome and factors that influence recovery) we analyzed the answer deemed most important by each respondent as described below.

For the most important answers on questions about domains of outcome (clinical, functional (independent living, social, daytime activities, other) and personal) the following analyses were performed;

- For each domain of outcome, we tested if the distributions of all answers across the answer categories (established with content analysis) differed significantly between respondent groups. Fisher-Freeman-Halton exact tests and pairwise Fisher’s exact tests with simulated p-values based on 10,000 replicates were used.
- For each answer category within each domain of outcome (e.g., positive symptoms for clinical recovery):
  - We tested if there was a difference in proportion of respondents that provided an answer in the investigated answer category between respondent groups.
    - First, Fisher-Freeman-Halton exact tests with simulated p-values based on 10,000 replicates were used to compare the three respondent groups together.
    - Second, for pairwise comparisons of respondent groups we used logistic regression with the answer category as dependent variable (e.g., if an answer in the answer category positive symptoms was given for clinical recovery yes/no) and the respondent groups (pairwise; service users, ICs or HCPs) as independent variables.
    - Third, to control for possible confounding aspects on which the respondent groups differed, the same logistic regressions were performed with addition of the following covariates: sex of respondent (male/female), level of education, (higher/lower), being spiritual (yes/no), sex of service user (male/female), primary psychiatric diagnosis (schizophrenia spectrum disorder (SSD) versus bipolar), treatment setting (no or outpatient versus inpatient) and recovery phase (late/early). For the data-analysis the recovery phases ‘being overwhelmed by’ and ‘struggling with’ the disease were merged into early phases of recovery and ‘living with’ and ‘living beyond’ the disease into ‘late’ phases of recovery. Because healthcare professionals reported on their client populations instead of on individual service users, the following parameters were not available for this group: years since first psychosis, sex of service user and diagnosis. (Supplementary material 1)
      A Bonferroni correction was performed for each domain of outcome, with α = 0.05 divided by the number of independent hypotheses. The number of independent hypotheses for each domain was calculated as 2 (for 3 dependent pairwise comparisons) * number of answer options.
  - Answer categories that contained less than 5 answers in total by all respondent groups together were excluded for analysis.

The same analyses as for domains of recovery were performed on the answers to questions about facilitators and barriers of recovery and personal strengths that contribute to recovery.

In an additional explorative analysis, we checked if the results changed when we analyzed all (maximum five) answers of respondents instead of only the one deemed most important by the respondent. The proportion of occurrence of answers in answer categories were visualized and compared to the results of analysis of most important answer only.

### Interpretation of Results

After data analysis, the results were presented to a research panel comprising six people with (a history of) psychosis, two family members of people with psychosis, four mental healthcare practitioners, and two researchers. Panel members first provided written feedback on the findings, identifying significant results and offering interpretations. Subsequently, three panel discussions were held to further analyze and interpret the results, ensuring a comprehensive evaluation from diverse perspectives.

## Results

### Respondent Characteristics

106 service users, 51 ICs and 69 HCPs completed the survey. The baseline characteristics of each respondent group with comparisons between respondent groups are summarized in Table 1 and Table 2. Of all respondents, 65% were female. Most ICs (69%) were parents and significantly older than service users and HCPs. Most HCPs were psychiatrist (26%), resident in psychiatry (14%) or nurse (32%). ICs reported mainly on service users classified with schizophrenia (55%), while in the service user group schizoaffective and bipolar disorders were common as well (12 and 33%, respectively). None of the service users and ICs reported (their loved one) being classified with a depression with psychotic features.

**Table 1:** Baseline characteristics of respondents

Variable	Service users (n = 106)	ICs (n = 51)	HCPs (n = 69)	Test statistic^a^	P
Mean age, years (SD)	44.4 (11.4)	59.4 (13.3)	44.1 (10.9)	F(2) = 34.15	** < 0.001**
Female sex, %	62.3	72.5	62.3	FE	0.41
Respondent born in the Netherlands, %	96.2	94.1	91.3	FE	0.22
Parents respondent born in Netherlands, %	85.8	88.2	88.4	FE	0.83
Educational level^b^, %				FE	** < 0.001**
Low + Medium	38.7	25.5	7.2		
High	61.3	74.5	92.8		
Being spiritual; yes, %	27.4	29.4	11.6	FE	**0.016**
Relationship with service user, %					
Parent		68.6			
Sibling		11.8			
Partner		11.8			
Other		7.8			
Frequency of contact, %					
Daily		39.2			
At least ones a week		51.0			
Several times a month or less		9.8			
Profession (%)^c^					
Nurse			33.3		
Psychologist			11.6		
Doctor			40.6		
Other			14.5		
Years work experience, %					
< 5 year			29.0		
5–10 years			13.0		
> 10 years			58.0		

Bold values indicate statistically significant differences (*p* < 0.05)

^a^Test statistics: ANOVA for comparison of means, Fisher Exact for comparison of proportions

^b^Level of education: Low = ISCED level 0–2; Medium = ISCED level 3–5, High = ISCED level 6–8. OECD, European Union, UNESCO Institute for Statistics. ISCED 2011 Operational Manual: Guidelines for Classifying National Education Programs and Related Qualifications. OECD Publishing; 2015

^c^The category ‘other professions’ contains five supported living workers, three social workers, one rehabilitation specialist and one psychomotor therapist

**Table 2:** Baseline characteristics of service users as reported by the different stakeholders groups

Variable	SUs (n = 106)	ICs (n = 51)	HCPs (n = 69)	Test statistic^a^	P
Mean age, years (SD)	44.4 (11.4)	40.5 (14.9)		F(1) = 3.38	0.07
Female sex, %	62.3	31.4		FE	** < 0.001**
First psychosis, %				FE	0.13
< 2 years ago	9.4	13.7			
2–5 years ago	19.8	13.7			
5–10 years ago	16.0	5.9			
> 10 years ago	54.7	64.7			
Unknown	0.0	2.0			
Diagnosis, %^b^				FE	**0.002**
Schizophrenia spectrum disorder	67.0	90.2			
Bipolar disorder	33.0	9.8			
Treatment setting, %^c,d^				FE	** < 0.001**
Inpatient treatment	0.9	13.7	*36.2*		
Living situation, %				FE	** < 0.001**
Independent	93.4	70.6			
Supported/sheltered housing	6.6	19.6			
Long-term admission	0.0	9.8			
Recovery phase, %^d^				FE	** < 0.001**
Overwhelmed by or struggling with disease	10.4	41.2	*76.8*		
Living with or beyond the disease	89.6	58.8	*23.2*		

Bold values indicate statistically significant differences (*p* < 0.05)

^a^Test statistics: ANOVA for comparison of means, Fisher Exact for comparison of proportions

^b^The question about diagnosis included all schizophrenia spectrum disorders, bipolar disorder, ‘other’ and ‘unknown’ as answering options. All answers of schizophrenia spectrum disorders, ‘other’ (service users 19.8%, ICs 11.8%) and ‘unknown’ (service users 5.7%, ICs 9.8%) were merged into ‘schizophrenia spectrum disorders’ in this table

^c^Treatment setting: 21 service users and people 7 ICs reported about were not under treatment. 10 HCPs were working in both outpatient and inpatient treatment settings, they were added to the ‘inpatient’ category

^d^Service users and ICs report about individual service users (themselves or their loved ones), while HCPs report about their total client population. To highlight this difference, data of HCP data are displayed in italics

‘No treatment’ or ‘outpatient treatment’ was the most common treatment setting for service users in all respondent groups (64–99%). Regarding recovery phase, most service user respondents were living with or beyond the disease (90%) while the ICs and HCPs report on service users they classified in early phases of recovery more frequently (41 and 57%, respectively).

### Domains of Recovery

#### Clinical Recovery

**Fig. 1:**
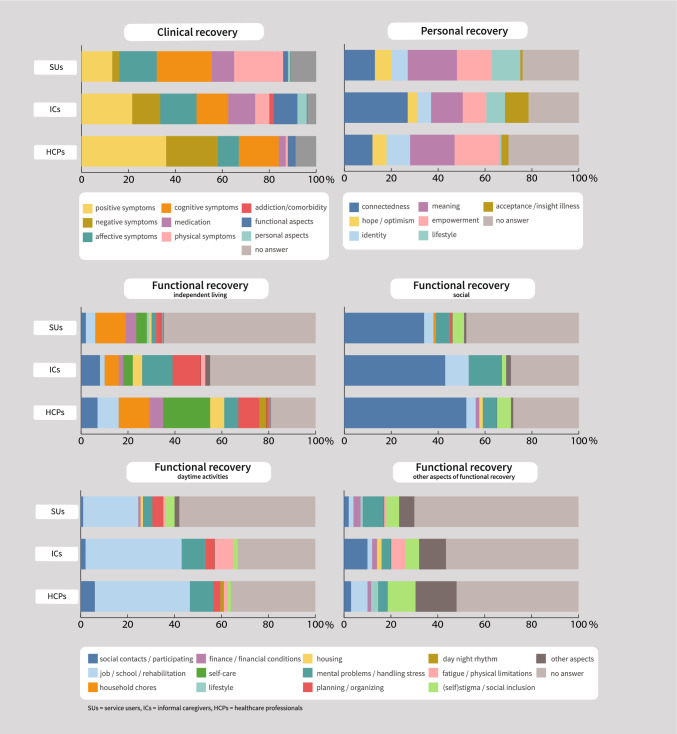
Most important aspects of recovery

With conventional content analysis ten answer categories were established (Fig. 1, Supplementary material 2). Service users deemed ‘cognitive symptoms’ (e.g., concentration problems and overstimulation) most often as most important answer category, followed by ‘physical symptoms’ (especially poor sleep and fatigue) and ‘affective symptoms’ (24%, 21% and 16%, respectively). ICs deemed ‘positive symptoms’ most often as most important answer category, followed by ‘affective’ (especially depressive symptoms and anxiety) and ‘cognitive symptoms’ (22%, 16% and 14%, respectively). HCPs deemed ‘positive symptoms’ (delusions, as well as hallucinations and formal thought disorders) most often as most important answer category, followed by ‘negative symptoms’ (especially social withdrawal and apathy) and ‘cognitive symptoms’ (36%, 22% and 17%, respectively).

Correction for covariates showed that ‘negative symptoms’ were mentioned by a higher proportion of HCPs than of service users (OR 6.39, 95%CI 1.32–37.57, p = 0.03) (Table 3). ‘Aspects of functional recovery’ as most important aspect of clinical recovery were mentioned by a higher proportion of ICs than of service users (OR 32.82, 95%CI 2.47–1752,86, p = 0.03) (Table 3). After Bonferroni correction for multiple testing (α = 0.004) these differences were no longer significant. An overview of the results of all statistical analysis is provided in Supplementary material 2.

**Table 3:** Differences in most important aspects of recovery between service users, informal caregivers and healthcare professional

	SU vs IC	IC vs HCP	SU vs HCP
Domains of recovery			
Clinical recovery	Aspects of functional recovery that influence clinical recovery (IC > SU) OR 32.82*		Negative symptoms: (HCP > SU) OR 6.39*
Functional recovery			
Independent living	Planning & organization (IC > SU) OR 8.80*		
Social			
Daytime activities	Fatigue and physical limitations: (IC > SU) OR 29.99*		
Other aspects of functional recovery			
Personal recovery		Connectedness (HCP < IC) OR 0.30*	
Priority of domains of recovery		Functional recovery (HCP < IC) OR 0.22*	Functional recovery (HCP < SU) OR 0.18*
Factors that influence recovery			
Facilitators of recovery		Healthcare (HCP < IC) OR 0.22**	Acceptance & insight (HCP > SU) OR 25.74* Lifestyle (HCP < SU) OR 0.05*
Barriers of recovery		Healthcare (HCP < IC) OR 0.13**	Healthcare (HCP < SU) OR 0.12*
Personal strengths that facilitate recovery		Social capacities (OR HCP < IC) OR 0.12*	

*p < 0.05, **p < 0.01. < means less often than, > means more often than

Only significant results of logistic regressions with covariate adjustment and without Bonferroni correction are displayed. An empty cell means that there were no significant differences between the groups for all investigated answer categories. A complete overview of the results for all investigated answer categories is displayed in Supplementary material 2

#### Functional Recovery

Conventional content analysis of the four subdomains of functional recovery (independent living, social functioning, daytime activities and other aspects of functional recovery) resulted in very similar answer categories. Therefore, we established the same 14 coding categories for these subdomains, facilitating easier comparison. All answers regarding intake or adherence to medication or treatment were coded as (treatment of) ‘mental problems/handling stress’. (Fig. 1, Supplementary material 2).

#### Independent Living

Service users deemed ‘household chores’ most often as most important answer category, followed by ‘finance/financial conditions’ and ‘self-care’ (13%, 5% and 5% respectively). ICs deemed ‘mental problems/handling stress’ most often as most important answer category, followed by ‘planning/organizing’ and ‘social contacts/participating’ (14%, 12% and 8% respectively). HCPs deemed ‘self-care’ most often as most important, followed by ‘household chores’, having ‘job/school/rehabilitation’ and ‘planning/organizing’ (20%, 13%, 9% and 9%, respectively).

Correction for covariates showed that ‘planning/organizing’ (e.g., having a daily routine) was mentioned by a higher proportion of ICs than of service users (OR 8.62, 95%CI 1.38–89.20, p = 0.04) (Table 3). After Bonferroni correction for multiple testing (α = 0.003) this difference was no longer significant. An overview of the results of all statistical analysis is provided in Supplementary material 2.

#### Social Functioning

All three respondent groups deemed ‘social contacts/participating’ most often as most important answer category (35% of service users, 43% of ICs, 52% of HCPs), followed by ‘mental problems/handling stress’ (6% of service users, 14% of ICs, 6% of HCPs). In addition, ICs mentioned ‘job/school/rehabilitation’ (10%) and service users and HCPs ‘(self)stigma/social inclusion’ as third most important (5% of service users, 6% of HCP). Correction for covariates showed no differences between groups (Table 3). An overview of the results of all statistical analysis is provided in Supplementary material 2.

#### Daytime Activities

‘Job/school/rehabilitation’ was deemed most often as most important answer category by all three respondent groups (by 24% of service users and 42% of ICs and HCPs), followed by ‘mental problems/handling stress’ (4% of service users, 10% of ICs and HCPs). Service users and HCPs mentioned ‘planning/organizing’ as third most important. (5% of service users and 3% of HCPs), while ICs mentioned ‘fatigue/physical limitations’ often (10%) as most important as well.

Correction for covariates showed ‘fatigue/physical limitations’ was mentioned as most important by a higher proportion of ICs than of service users (OR 29.99, 95%CI 2.51–1173.66, p = 0.02) (Table 3). After Bonferroni correction for multiple testing (α = 0.004) these differences were no longer significant. An overview of the results of all statistical analysis is provided in Supplementary material 2.

#### Other Aspects of Functional Recovery

Service users deemed ‘mental problems/handling stress’ most often as most important answer category (9%). ICs deemed ‘social contacts/participating’ most often as most important answer category, followed by ‘fatigue/physical limitations’ (10% and 6% respectively). HCPs deemed ‘(self)stigma/social inclusion’ most often as most important (12%), and this answer category was deemed important by service users and ICs as well (both 6%). In addition, HCPs frequently mentioned ‘job/school/ rehabilitation’ as most important (7%). Answers that could not be coded in other answer categories (category ‘other aspects’) were frequently provided by all respondent groups (17% of HCPs, 12% of ICs and 7% of service users). In this ‘other aspects’ category aspects of meaning in life, hope and perspective, and professional mental healthcare were deemed important by all respondent groups.

Correction for covariates showed no differences between groups (Table 3). An overview of the results of all statistical analysis is provided in Supplementary material 2.

#### Personal Recovery

Because conventional content analysis resulted in too many, detailed categories, the CHIME framework (Connectedness, Hope and optimism about the future, Identity, Meaning in Life, Empowerment—giving the acronym CHIME) was used as a starting point for coding categories (Leamy et al., 2011). The importance of CHIME is widely endorsed in recovery literature (van Weeghel et al., 2019). Studies investigating the applicability of the CHIME framework from a service users perceptive have supported the category structure (Bird et al., 2014; Skar-Fröding et al., 2021; Stuart et al., 2017). Our data did not provide a basis for further subdividing the CHIME criteria. 13% of answers could not be categorised according to the CHIME categories. With conventional content analysis, the categories ‘lifestyle’, ‘acceptance of/insight into illness’ were added. This resulted in a total of seven coding categories (Fig. 1, Supplementary material 2).

All three respondent groups deemed the same three answer categories most often as most important, namely ‘connectedness’ (13% of service users, 27% of ICs and 12% HCPs), ‘meaning (of life)’ (21% of service users, 14% of ICs and 19% of HCPs) and ‘empowerment’ (15% of service users, 10% of ICs, 19% of HCPs). In addition, service users deemed ‘lifestyle’ (e.g., balance, relaxation and being physically fit) often as most important (12%), ICs ‘acceptance of/insight into illness’ (10%) and HCPs ‘identity’ (10%).

Correction for covariates showed that ‘connectedness’ was mentioned as most important by a lower proportion of HCPs than of ICs (OR 0.30, 95%CI 0.09–0.91, p = 0.04) (Table 3). After Bonferroni correction for multiple testing (α = 0.004) this difference was no longer significant. An overview of the results of all statistical analysis is provided in Supplementary material 2.

#### Domains of Outcome

Service users and ICs equally emphasized the importance of clinical, functional, and personal recovery aspects. HCPs deemed aspects of clinical recovery most often as most important (29%), followed by aspects of personal recovery (22%).

Correction for covariates showed that aspects of functional recovery were mentioned as most important by a lower proportion HCPs than of service users (OR 0.18, 95%CI 0.03–0.78, p = 0.04) and of ICs (OR 0.22, 95%CI 0.06–0.70, p = 0.02) (Table 3). After Bonferroni correction (α = 0.006) for multiple testing this difference was no longer significant. An overview of the results of all statistical analysis is provided in Supplementary material 2.

### Factors that Influence Recovery

#### Facilitators of Recovery

**Fig. 2:**
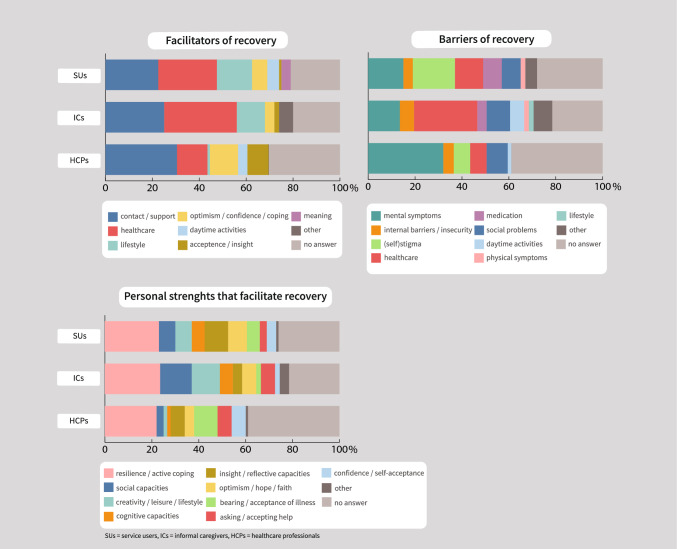
Factors that influence recovery

With conventional content analysis, eight coding categories were established (Fig. 2, Supplementary material 2).

All three respondent groups deemed ‘contact/support’ (23% service users, 25% ICs, 30% HCPs) and ‘healthcare’ (35% service users, 31% ICs, 13% HCPs) often as most important. In addition, service users and ICs often mentioned ‘lifestyle’ as most important (15% and 12%, respectively), while HCPs more often mentioned ‘optimism/confidence/coping’ (12%) and ‘acceptance/insight’ (9%).

Correction for covariates showed that ‘acceptance/insight’ was mentioned as most important by a higher proportion of HCPs than of service users (OR 25.74, 95%CI 1.69–840.92, p = 0.03) (Table 3). ‘Lifestyle’ was mentioned by a lower proportion of HCPs than of service users (OR 0.05, 95%CI 0.00–0.52, p = 0.04) (Table 3). ‘Healthcare’ was mentioned by a lower proportion of HCPs than of ICs (OR 0.22, 95%CI 0.07–0.65, p = 0.01) (Table 3). After Bonferroni correction for multiple testing (α = 0.004) these differences were no longer significant. An overview of the results of all statistical analysis is provided in Supplementary material 2.

#### Barriers of Recovery

With conventional content analysis, 10 coding categories were established (Fig. 2, Supplementary material 2).

Service users most often mentioned ‘(self)stigma’ as most important barrier for recovery (18%), ICs ‘healthcare’ (27%) and HCPs ‘mental symptoms’ (32%). Correction for covariates showed that ‘healthcare’ was mentioned by a lower proportion of HCPs than of ICs (OR 0.13, 95%CI 0.03–0.46, p = 0.003) and service users (OR 0.12, 95%CI 0.02–0.63, p = 0.02) (Table 3). After Bonferroni correction for multiple testing (α = 0.004) the difference between ICs and HCPs remained significant. An overview of the results of all statistical analysis is provided in Supplementary material 2.

#### Personal Strengths that Facilitate Recovery

With conventional content analysis, ten coding categories were established (Fig. 2, Supplementary material 2).

All respondent groups most often mentioned ‘resilience/active coping’ as the most important personal strength that facilitates recovery.

Correction for covariates showed that ‘social capacities’ were mentioned by a lower proportion of HCPs than of ICs (OR 0.12, 95%CI 0.01–0.71, p = 0.03) (Table 3). After Bonferroni correction for multiple testing (α = 0.003) this difference was no longer significant. An overview of the results of all statistical analysis is provided in Supplementary material 2.

### Explorative Analysis of all Provided Answers

We checked if the proportions of answer categories for the domains of outcome changed when we used all answers instead of only the ones deemed most important by the respondent for analysis. Results are displayed in Supplementary material 3. For clinical recovery, we found that the strong prioritization on positive symptoms among HCPs became smaller when analyzing all answers. Because of that, the priorities of HCPs became more similar to those of service users and ICs in the analysis of all answers. For independent living, we found that in all respondent groups financial conditions and lifestyle factors were mentioned relative more frequently when analyzing all answers. For the other domains of recovery and factors that influence recovery, the results of the analysis of all answers were in line with the results of the analysis of most important answer only.

## Discussion

In this exploratory study, we investigated the priorities regarding the recovery outcomes of psychotic disorders from the perspectives of service users, informal caregivers, and healthcare professionals. In addition, we looked at factors that according to these groups promote or hinder recovery. While we identified several differences among stakeholder groups, commonalities prevailed.

### Summary of Results

All stakeholder groups recognized the importance of the pursuit of both clinical, functional, and personal recovery. However, service users and ICs appeared to assign equal value to these domains, whereas HCPs prioritized clinical recovery, followed by personal recovery. Concerning functional recovery, stakeholder groups generally agreed on the most crucial aspects. Notably, some disparities emerged between ICs and service users. ICs prioritized ‘planning/organizing’ for independent living and ‘fatigue/physical limitations’ for daytime activities more frequently than service users. In terms of personal recovery, the only difference between stakeholder groups was the prioritization of ‘connectedness’. ICs considered ‘connectedness’ as most important, prioritizing it more often than service users.

The primary differences in the priorities of factors that influence recovery among stakeholder groups were related to the role of ‘mental healthcare’. ICs prioritized ‘healthcare’ more often than HCPs as a facilitator of recovery, and both service users and ICs identified problems with ‘healthcare’ more frequently as a significant barrier to recovery compared to HCPs. In addition, service users more often prioritized ‘lifestyle’ as a facilitator of recovery compared to HCPs, and ICs more often emphasized ‘social capacities’ as a facilitator of recovery compared to HCPs. Finally, HCPs emphasized ‘acceptance/insight’ more often than service users as a facilitator of recovery.

### Interpretation of Results

Regarding prioritization of domain of outcome, our findings suggest smaller differences between stakeholder groups than other studies (Karow et al., 2012; Sood et al., 2022), but similar trends persist. These trends also appear in the priorities of factors that might influence recovery where HCPs more often prioritize clinical aspects (‘acceptance/insight’), while ICs and service users more often prioritize functional and personal aspects (‘healthcare’, ‘lifestyle’ and ‘connectedness’). Results regarding the most important aspects of functional recovery indicate that our division into subdomains is somewhat artificial, as the same aspects are mentioned across most subdomains. This can be explained by the fact that functioning well in one subdomain (e.g., social) contributes to functioning well in another subdomain (e.g., daytime activities). Above all, and in line with previous research, the answers regarding functional recovery expressed the need to be self-sufficient (Sood et al., 2022). The results of the analysis of all answers were mostly in line with the results of the analysis of most important answer only. The observed differences in responses suggest that respondents adhered to the instruction to prioritize their answers, placing what they deemed most important first. Upon analyzing all answers, it became apparent that responses were more evenly distributed across various categories, and respondents included more conditions deemed necessary by them for recovery.

Our research revealed some important differences in recovery priorities among stakeholder groups. An important aspect that might contribute to these differences are the different roles and viewpoints of the stakeholders involved. In our study, service users most likely reported from their own perspective while ICs and HCPs most likely incorporated both their own and the service user perspective in their answers. For instance, HCPs might emphasize aspects of clinical recovery due to their sense of responsibility for the safety of service users and their surrounding environments, particularly when the psychotic symptoms lead to behaviors that pose serious harm to the service user or others. Furthermore, the treatment options available to HCPs often target symptomatic relief. The emphasis on ‘Connectedness’ by informal caregivers might reflect their own strong desire to maintain an appropriate relationship with those they care fore (Yasuma et al., 2022). Additionally, worries about the small social network of those they care for could stem from their own social needs, excessive concern, or fears about the future well-being of the service users in their absence (Sood et al., 2022). In contrast, service users may not always experience a lack of connectedness with loved ones or having a small social network as (most important) problems themselves.

A second aspect that might contribute to differences in prioritized answers are differences in the abstraction levels of answers between stakeholders. Regarding independent living service users seem to focus on practical issues (‘household chores’) while informal caregivers addressed possible underlying causes (‘planning/organizing’ and ‘mental problems/handling stress’). This finding is in line with a study on the role of spirituality on recovery in people with schizophrenia, that showed that the answers of service users were factual and concrete compared to the more abstract and cognitive answers of HCP (Ho et al., 2016). A possible explanation might be from a strength—and ability focus perspective that service users have a focus on managing their specific problems in daily life and functioning. However, the lack of abstraction in answering may also be caused by aberrant abstraction in service users with schizophrenia spectrum disorders (Ho et al., 2016; Rosen et al., 2021).

A third aspect that might contribute to differences in prioritized answers is the (self)stigma on (people with) psychosis. Negative experiences related to a lack of support or stigmatization of service users in mental healthcare were more often mentioned as barriers of recovery by service users and informal caregivers than by professionals. This result is in line with qualitative research with service users, ICs and HCPs on the barriers in the process of shared decision making (Kokanović et al., 2018; Villena-Jimena et al., 2023). Several studies have indicated that HCPs, despite their expertise and training, are not immune to the stigmatizing beliefs and prejudices that are commonly associated with mental illness (Lauber et al., 2004). These attitudes may not always be overt or intentional, and professionals might not be aware of their own biases (Kopera et al., 2015). Perceived stigma of HCPs by service users, characterized by negative affective reactions and perceived social distance, are associated with internalized stigma and disempowerment (Wang et al., 2018).

Fourth, we should be aware of diagnostic overshadowing as a factor contributing to differences in priorities between stakeholder groups. In diagnostic overshadowing, normal desires, problems, or opposing viewpoints are misinterpreted through the lens of a person’s mental illness (Shefer et al., 2014). This carries the risk that behavior of service users deviating from social norms or one’s own frame of reference may be too quickly interpreted as a sign of illness by healthcare professionals or informal caregivers.

### Strengths and Limitations

The study is one of the first larger studies comparing perspectives of both service users with (a history of) psychosis, ICs and HCPs on aspects of recovery. All stakeholder groups participated in the design of the study and interpretation of results. Compared to the often used focus groups and interviews used in this research field, the use of anonymous surveys might have reduced the risk of being influenced by others and giving desirable answers.

The study also has several limitations. First, the use of an open online questionnaire in Dutch poses a risk of selection bias. The large majority of service user respondents in our study lived independently, received outpatient treatment and considered themselves as living with of beyond the illness. People with severe clinical symptoms or low levels of social and community participation might be underrepresented because the call to participate might not have reached them, they did not have access to a computer or where not able to complete the questionnaire. Conversely, overrepresentation of respondents facing healthcare system challenges might skew results, as those with issues may be more willing to participate to voice their concerns. With 90% of participating ICs reporting weekly contact with their loved one with psychosis, closely involved ICs might be overrepresented. The survey being in Dutch excludes non-Dutch speakers, potentially introducing a cultural bias, as fewer than 10% of respondents in all stakeholder groups were born outside the Netherlands, compared to 15% of the general Dutch population (CBS, 2023). Previous research about recovery in people with black and minority ethnic backgrounds identified a greater emphasis on spirituality, stigma, culture-specific factors and collectivist notions of identity (Leamy et al., 2011). Overall, the risk of selection bias might limit the generalizability of results towards the more severely affected service users and their family members and non-Western cultures.

Second, there is no consensus on the definition of recovery, which may influence responses, as recovery can for instance be seen either as an expected outcome or an experiential process (Leonhardt et al., 2017).

Third, our study did not involve triads of service users, ICs, and HCPs, which does not make it possible to compare perspectives on the same service user.

Fourth, our questionnaire did not have forced answering categories and many respondents did not answer all questions, resulting in large ‘no answer’ categories in our results. We do not know why respondents did not answer a question making it difficult to interpret the findings. Possible reasons to not answer a question are that respondents did not experience any challenges on a specific domain or had no ideas about the domains, facilitators or barriers of recovery. Respondents did not appear to skip more questions as the survey progressed, making fatigue an unlikely explanation for missing responses.

A last limitation is the use of an existing theory in directed content analyses, as we did with the widely endorsed CHIME framework for the domain of personal recovery. This introduces an (informed) bias towards the existing theory when coding the data. To mitigate this bias, we checked whether the data supported the subdivision of CHIME categories and added two additional categories for responses that could not be coded within the existing CHIME framework. However, it remains possible that a purely inductive approach might have yielded somewhat different results.

### Clinical Implications

While commonalities were evident, our study identified noteworthy distinctions in how various stakeholder groups prioritize recovery domains. Our primary message for all stakeholders is to acknowledge and respect potential differences in perspectives on recovery goals.

Facilitating an open dialogue about these varying viewpoints is imperative to prevent miscommunication. The identified differences in perspectives underscore the importance of fostering collaboration among triads of service users, informal caregivers (ICs), and healthcare professionals (HCPs) at multiple levels—research, policy, healthcare organizations and individual patient care and support (Zomer et al., 2020). To ensure representation at the policy and organizational levels, service user and informal caregiver perspectives can be effectively voiced through service user and family associations and/or experts with lived experience. On an individual service user level, adhering to a recovery-oriented healthcare approach requires placing the service users’ goals at the core of treatment. Nonetheless, service users may require psycho-education, assistance, and support from their informal caregivers and healthcare professionals to make informed decisions (Kokanović et al., 2018). Innovations in mental healthcare, like Active Recovery in the Triad (ART), Resource ACT (RACT) and Peer-supported Open Dialogue (POD), respond to the need to improve communication between service users, ICs and HCPs (Razzaque & Stockmann, 2016; Tjaden et al., 2020; Zomer et al., 2020).

A second lesson learned from this study is that stakeholders use different language to express their ideas about recovery and seem to think about recovery in different levels of abstraction. Service users described concrete personal circumstances and goals, ICs described the necessary conditions to achieve goals and HCPs described recovery in general, using medical terminology. For example, when a service user describes problems with cooking, his IC describes problems with planning and his HCP describes cognitive problems they might all refer to the same problem but also to completely different problems. This emphasizes the importance of clear and concrete communication between stakeholders. Peer-supported open dialogue explicitly involves experts by experience to advance the dialogue between service user, ICs and HCPs and to create a common understanding of a presented difficulty through shared language (Fedosejevs et al., 2023).

In the collaboration between stakeholders on an organizational level and in daily practice, differing viewpoints on the importance of healthcare as a factor influencing recovery should be acknowledged and discussed. These differing viewpoints may lead to varying perceptions of healthcare quality and may result in differences in the perceived scope of influence and perceived responsibilities of stakeholders. It is important to discuss these varying perspectives to ensure effective collaboration. Further participatory research is needed to explore ways to enhance communication and collaboration among stakeholders in mental healthcare.

## Supplementary Information

Below is the link to the electronic supplementary material.

Supplementary file 1 (PDF 38 KB)

Supplementary file 2 (XLSX 34 KB)

Supplementary file 3 (PDF 323 KB)
