# Supplementary material for: In Pursuit of Recovery: A Comparative Study of Stakeholder Perspectives on Outcomes of People with Psychosis
Source: Community Ment Health J. 2024 Dec 10;61(2):300–13. doi: 10.1007/s10597-024-01399-9 (PMC11772473; doi:10.1007/s10597-024-01399-9)
Supplement: Supplementary file 1 — (PDF 38 KB) [file 10597_2024_1399_MOESM1_ESM.pdf]

Online Resource 1 Independent variables in logistic regression analyses with covariates

	**Service users vs Informal caregivers**	**Informal caregivers vs Healthcare professionals**	**Service users vs Healthcare professionals**
Covariates:			
1. Respondent group			
2. Sex of respondent (male/female)	X	X	X
3. Level of education, (higher)	X	X	X
4. Being spiritual (yes)	X	X	X
5. Sex of service user	X	-	-
6. Primary psychiatric diagnosis (schizophrenia spectrum disorder (SSD)	X	-	-
7. Treatment setting (outpatient)	X	X	X
8. Recovery phase (late)	X	X	X

Not all respondent- and service user characteristics were analyzed as covariates for various reasons. Age of both respondents and service user was not added as covariate because the older age of informal caregivers was likely to be related to the fact that most informal caregivers were parents of service users with psychotic vulnerability. Migration background and years since first psychosis were not analyzed as a covariates because we had to limit the amount of added variables in the model to avoid overfitting and because there were no significant differences between respondent groups. Although there were significant differences between groups regarding living situation, we did not add these as covariate because of high correlation (0.58) with treatment setting.
