# Supplementary material for: In Pursuit of Recovery: A Comparative Study of Stakeholder Perspectives on Outcomes of People with Psychosis
Source: Community Ment Health J. 2024 Dec 10;61(2):300–13. doi: 10.1007/s10597-024-01399-9 (PMC11772473; doi:10.1007/s10597-024-01399-9)
Supplement: Supplementary file 3 — (PDF 323 KB) [file 10597_2024_1399_MOESM3_ESM.pdf]

Online Resource 3 Explorative analysis of all answers

Domains of recovery

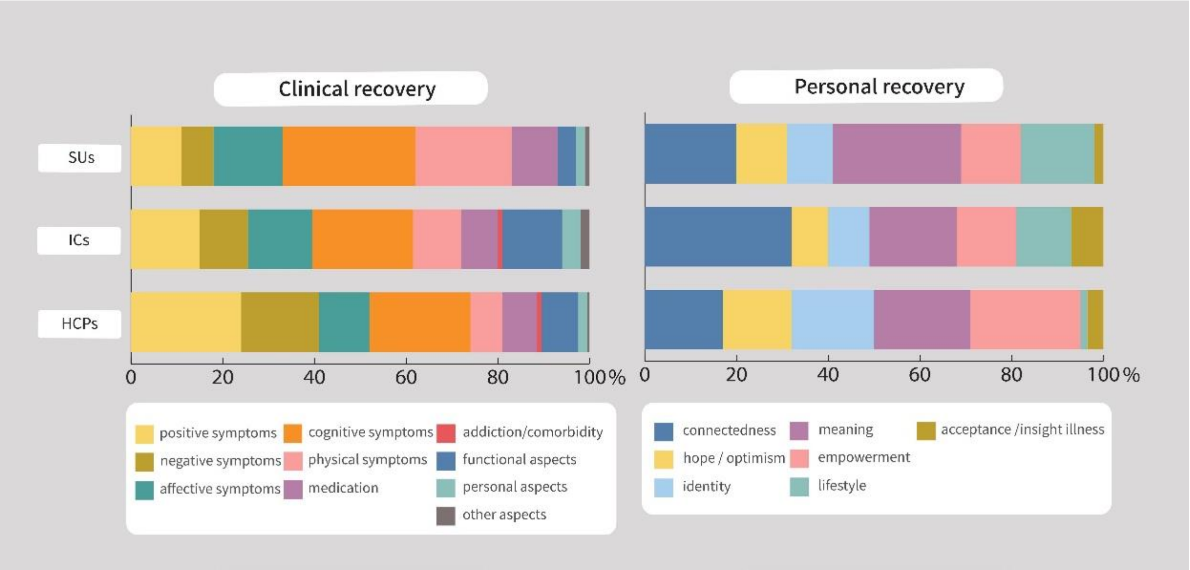

Clinical recovery: results for service users based on a total of 300 answers by 94 respondents, for ICs based on 190 answers by 49 respondents and for HCPs based on a total of 282 answers by 63 respondents.

Personal recovery: results for service users based on a total of 279 answers by 81 respondents, for ICs based on 138 answers by 40 respondents and for HCPs based on a total of 198 answers by 48 respondents.

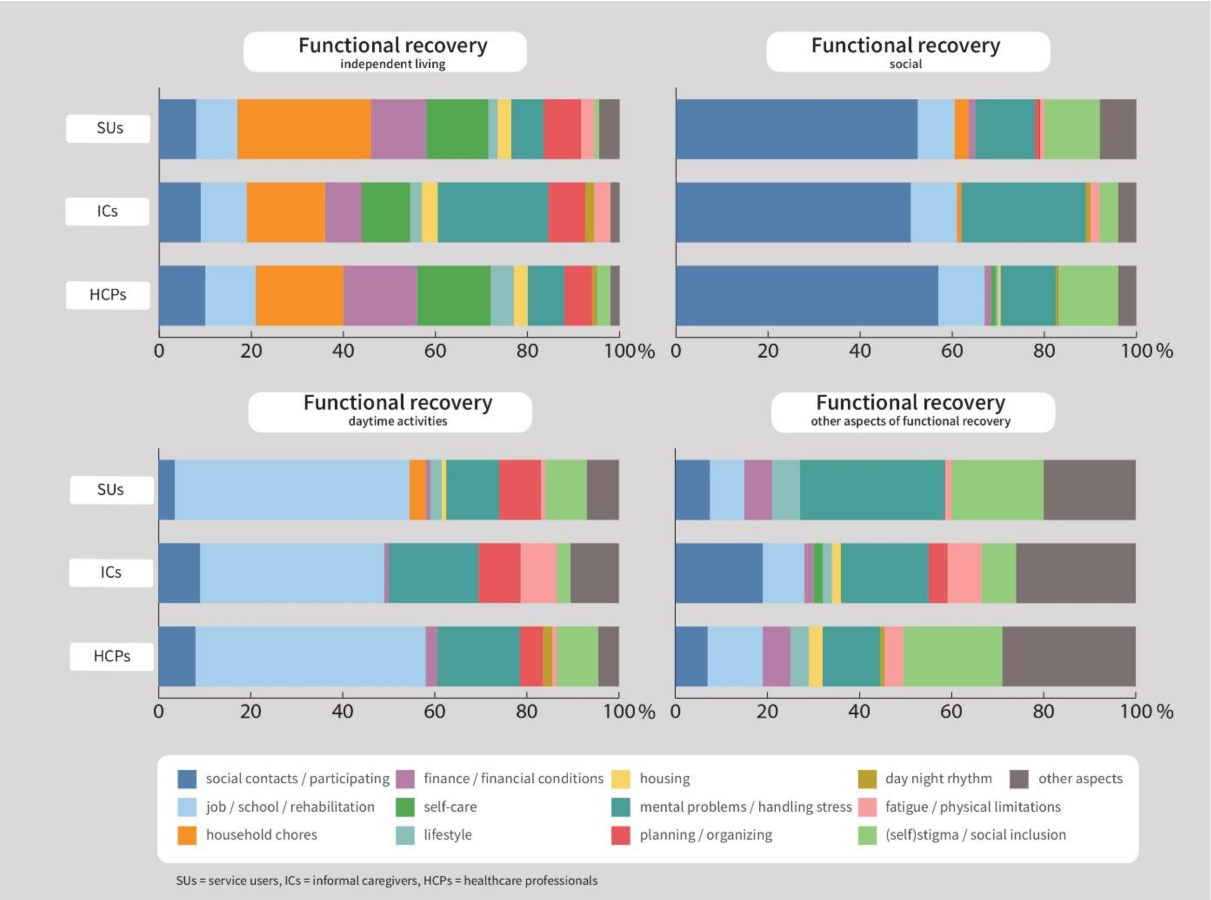

Functional recovery: independent living: results for service users based on a total of 90 answers by 37 respondents, for ICs based on 88 answers by 28 respondents and for HCPs based on a total of 210 answers by 56 respondents.

Functional recovery: social: results for service users based on a total of 116 answers by 55 respondents, for ICs based on 82 answers by 36 respondents and for HCPs based on a total of 184 answers by 50 respondents.

Functional recovery: daytime activities: results for service users based on a total of 87 answers by 44 respondents, for ICs based on 77 answers by 34 respondents and for HCPs based on a total of 151 answers by 44 respondents.

Functional recovery: other aspects of functional recovery: results for service users based on a total of 66 answers by 32 respondents, for ICs based on 53 answers by 22 respondents and for HCPs based on a total of 102 answers by 33 respondents.

Factors that influence recovery

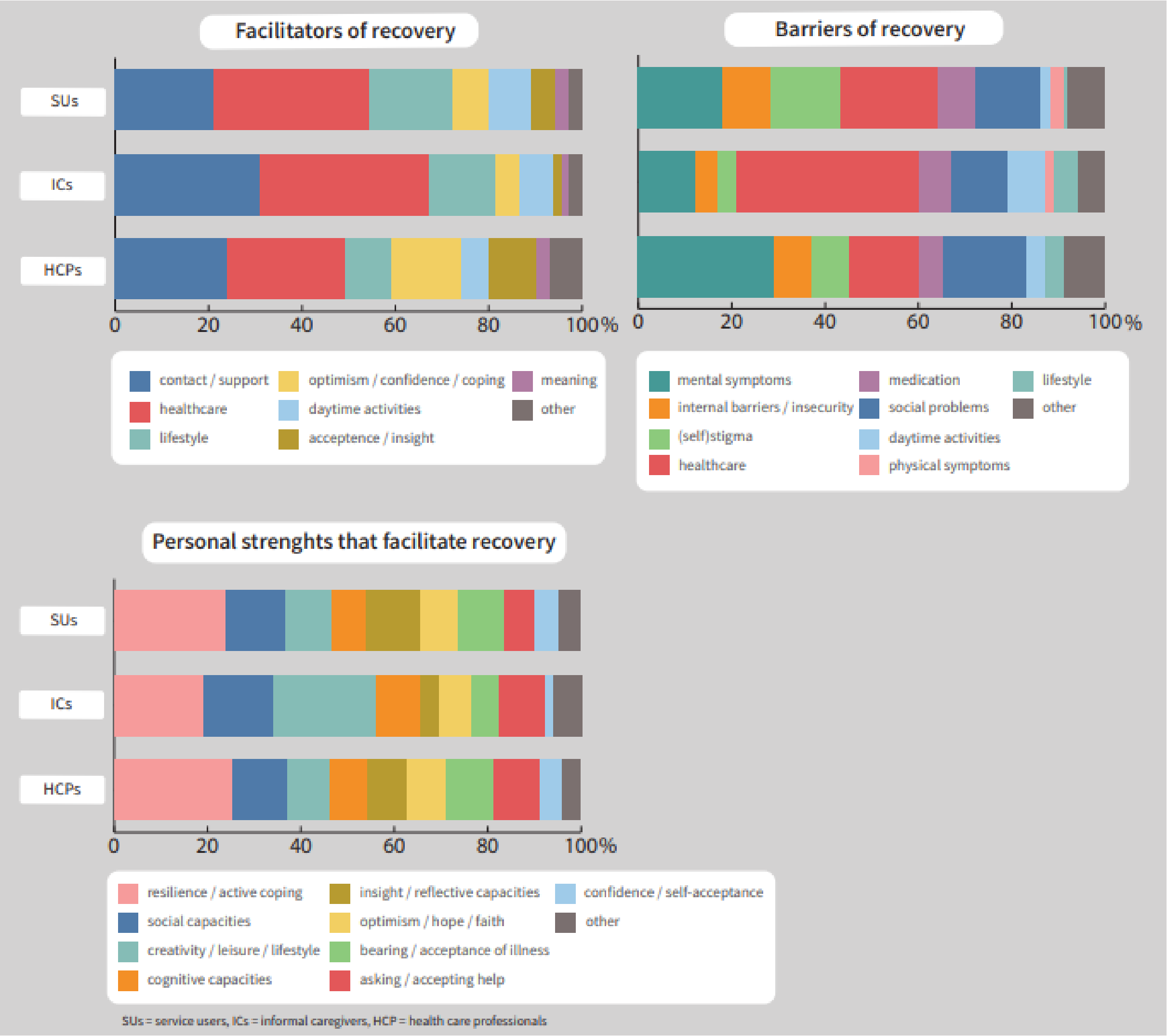

Facilitators of recovery: results for service users based on a total of 316 answers by 84 respondents, for ICs based on 117 answers by 41 respondents and for HCPs based on a total of 210 answers by 48 respondents.

Barriers of recovery: results for service users based on a total of 238 answers by 76 respondents, for ICs based on113 answers by 40 respondents and for HCPs based on a total of 189 answers by 42 respondents.

Personal strengths that facilitate recovery: results for service users based on a total of 280 answers by 78 respondents, for ICs based on 125 answers by 40 respondents and for HCPs based on a total of 175 answers by 42 respondents.

In Pursuit of Recovery; A Comparative Study of Stakeholder Perspectives on Outcomes of People with Psychosis. Community Mental Health Journal.
